# Factors affecting family medicine programmes in Sub-Saharan Africa: a narrative review of recent literature

**DOI:** 10.4314/gmj.v56i4.10

**Published:** 2022-12

**Authors:** Matthew L Davies, Penelope K Ellis, Akin Moses, Henry Lawson, Albert Akpalu, Richard W Walker

**Affiliations:** 1 Population Health Sciences Institute, Newcastle University, Baddiley-Clark Building, Newcastle upon Tyne NE2 4AX. United Kingdom; 2 Northumbria Healthcare NHS Foundation Trust, North Tyneside General Hospital, Rake Lane, North Shields, Tyne and Wear NE29 8NH. United Kingdom; 3 National Hospital Abuja, Department of Family Medicine, Abuja, Federal Capital Territory, NG; 4 Ghana College of Physicians and Surgeons, Family Medicine Unit, Dept of Community Health, Accra, Greater Accra, GH; 5 Korle Bu, Medicine, PO Box 4236, Accra, GH Box 77

**Keywords:** Family medicine, practice, Sub-Saharan Africa

## Abstract

**Objective:**

To identify the factors enabling and limiting family medicine (FM) programmes in Sub-Saharan Africa (SSA).

**Design:**

A narrative review was conducted by searching a variety of databases. Papers focusing on the training, deployment, or contribution to healthcare systems of doctors with postgraduate training in FM in SSA, published in peer-reviewed journals from 2015 onwards and in English language were included. Included papers underwent qualitative analysis.

**Results:**

Seventy-one papers were included in the review. 38% focussed on South Africa, while papers focussing on FM in a further 15 countries in SSA were identified. Key factors enabling FM programmes are support from key stakeholders, recognition of family practitioners (FP) as specialists, international collaboration, and dedicated FPs. Key factors limiting FM programmes are a lack of sufficient and well-trained faculty, inappropriate training settings, higher rates of trainee attrition, lack of FM in undergraduate curriculums, lack of career pathways, inappropriate deployment, and a lack of a critical mass.

**Conclusions:**

Support from national stakeholders, the recognition of FPs as specialists, and sustainable international collaboration promote FM programmes. The absence of a defined role within the healthcare system, low numbers of FM faculty, a poor presence in undergraduate curriculum, high attrition rate of trainees and the lack of a critical mass limit FM programmes. The standardisation of the role of FM and the implementation of undergraduate and postgraduate FM programmes with national and international collaboration could enable FM to reach a critical mass and realise its full potential in strengthening primary healthcare in SSA.

**Funding:**

None declared

## Introduction

Sub-Saharan Africa (SSA) faces significant challenges in providing timely, accessible, high-quality healthcare.^1^,^2^ A 2017 World Health Organisation (WHO) report identified that SSA has the lowest healthcare service coverage of all regions worldwide.^3^ Only three countries in the region have met the target of spending 15% of GDP in health, and there is a significant shortage of healthcare workers.^4^

Thirty-one (57%) countries in SSA meet the criteria for a critical shortage of healthcare workers; under 22.8 health care professionals per 10,000 population.^5^ Despite many SSA countries expanding healthcare programmes, the deficit in healthcare coverage is predicted to worsen due to increasing population sizes.^5^,^6^

To improve global access to healthcare, the United Nations has committed to achieving universal health coverage (UHC) by 2030 (Sustainable Development Goal 3.8).^7^ The WHO defines UHC as “healthcare that ensures all people and communities can use the promotive, preventive, curative, rehabilitative and palliative health services they need, of sufficient quality to be effective, while also ensuring that the use of these services does not expose the user to financial hardship”.^1^ High quality primary health care (PHC) has been identified as “the engine for UHC”. An effective healthcare system that provides high quality PHC and avoids a centralised, vertical healthcare model is the most comprehensive and cost-effective method to achieve UHC.^7^–^9^

In response to the call to strengthen PHC services, many SSA countries have established Family Medicine (FM) programmes. FM is an expanding population-orientated discipline that seeks to supply “expert generalists” that provide community health care.^10^,^11^ The 2009 Rustenburg consensus identified the roles of a Family Practitioner (FP) as “a comprehensive set of skills adapted to the circumstances, local needs, available resources, facilities and the competency and limitations of the practitioner”.^12^ In practise the role of FPs in SSA is largely varied, from supporting district hospital community outreach to being an integral member of PHC in the community. 13 Despite the range of roles, the importance of FPs in strengthening PHC has been identified.^14^,^15^

The COVID-19 pandemic has caused significant disruption to global healthcare services, widened inequalities, and emphasised the disparity in global UHC coverage.^1^ It is now more critical than ever to improve the provision of UHC. FM programmes could play a key role in strengthening PHC, supporting healthcare services and have a key role in future pandemics.^16^–^18^ A recent scoping review by Flinkenflogel et al has identified how FM is implemented, the impact, strengths and weaknesses, and the role of FPs in Africa. This narrative review seeks to build on this work and identify the current factors that affect FM programmes.^11^ By understanding these factors FM programmes can be improved to support the effective deployment of FPs and the provision of UHC.

## Methods

The narrative review was performed to investigate the factors enabling and limiting FM programmes in SSA. PubMed, EMBASE, ERIC and Global Health databases were searched for relevant literature. The medical subject heading (MeSH) and search terms used to search are shown in Table 1. The search was restricted to papers published from 2015 onwards to retrieve sufficient literature and ensure that our findings reflected the current status of postgraduate FM in SSA.

**Table 1:** MeSH and search terms used to identify litera-ture for inclusion in the review

Search Fields		Search Term
All		Africa OR African OR Country Name*
Title or Abstract	AND	Family Medicine OR Family Practice OR Family Physician OR Family Doctor OR General Practitioner

* One of the 48 countries in SSA using The World Bank definition

The searches were carried out on 18^th^ November 2021. Specific inclusion and exclusion criteria are listed in Table 2.

**Table 2:** Inclusion and exclusion criteria for papers included within the review

Should be an article published in a peer-reviewed journal Should focus wholly or significantly on the training, deployment, or contribution to healthcare systems of doctors with postgraduate training in Family Medicine Should focus wholly or significantly on one or more countries in SSA (defined by The World Bank) Should be written in English Should not focus on the management of a specific condition (but papers examining the contribution of postgraduate, family medicine doctors to a specific area of medicine – e.g., obstetrics or surgery – are included) Should not focus on use of guidelines Should not focus on healthcare funding models Should not focus on commenting on or be written in response to another piece of published work (e.g., letters to an editor) Should not be a review of previously published literature Should not focus on specific approaches to teaching (e.g., problem-based curricula) or use of spe-cific teaching aids (e.g., maintenance of e-portfolios).

**Figure 1:**
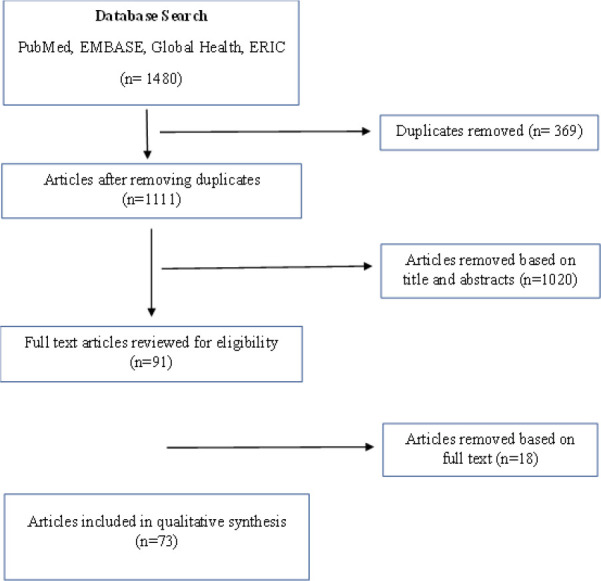
Literature selection strategy

Following initial identification of papers, any duplicates were identified and removed. Papers were initially selected using the title and abstract. A total of 91 full text papers were read by two authors, MLD and PKE. Any papers that did not meet the inclusion criteria were excluded. If authors disagreed on the inclusion or exclusion of a paper, the full article was reviewed, and the authors assessed and discussed the paper in terms of the inclusion and exclusion criteria before coming to a final agreement. Figure 1 summarises the process for identifying the papers included in the final review.

Following inclusion, papers were analysed for data addressing the following research questions.

1. What are the major factors enabling FM in SSA?
2. What are the major factors limiting FM in SSA?

Qualitative analysis of the included papers was performed. Both MLD and PKE coded the data according to the presence of information addressing each of the research questions. Thematic codes were identified within the initial coding and were discussed by both these authors. Data relevant to these themes were labelled. The coded information was summarised for inclusion in the final analysis and final analysis was performed by an inductive approach.

## Results

Seventy-three papers were included in the narrative review. Twenty-eight papers were focussed on South Africa (38.4%). Eight papers concentrated on SSA, five each on Ethiopia and Nigeria, four on Kenya, three each on Botswana, Ghana, Lesotho and Sudan, two on Zambia, and one each on Liberia, Malawi, Mali, Namibia, Sierra Leone, Tanzania, Uganda and Zimbabwe. One paper focused on both Rwanda and South Africa. The African Journal of Primary Health Care and Family Medicine was the most popular journal for publication of papers, with 28 (38.4%). This was followed by South African Family Practice with 17 papers (23.3%). The remainder of the papers were published in a further 19 journals.

**Figure 2:**
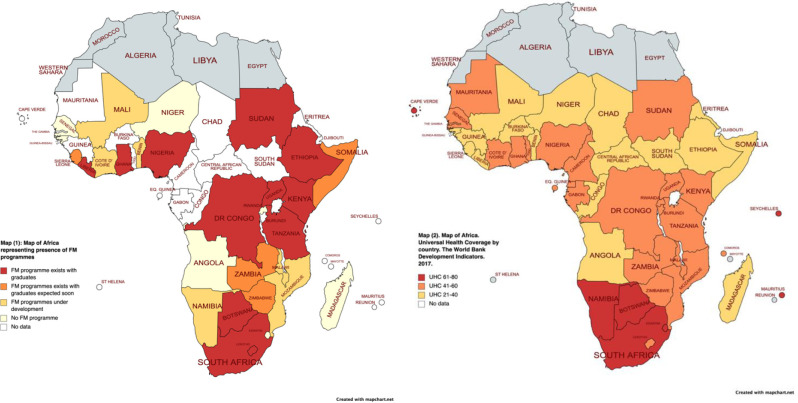
Map 1(Left) displays countries in SSA with the differing presences of FM programmes per country. 10,19–54 Map 2(Right) displays UHC per country in SSA. Notes to Figure 2 Map (1): Map of Africa representing presence of FM programmes. Red = FM Programme Exists with Graduates, Orange = FM Programme Exists with Graduates Expected Soon, Yellow = FM Programme Under Development, White = No FM Programme, Grey = No data Map (2): Map of Africa. Universal Health Coverage by country. Data from the World Bank Development Indicators. Coverage of essential health services (defined as the average coverage of essential services based on tracer interventions that include reproductive, maternal, newborn and child health, infectious diseases, non-communicable diseases and service capacity and access, among the general and the most disadvantaged population). The indicator is an index reported on a unitless scale of 0 to 100, which is computed as the geometric mean of 14 tracer indicators of health service coverage. The tracer indicators are as follows, organized by four components of service coverage: 1. Reproductive, maternal, newborn and child health 2. Infectious diseases 3. Noncommunicable diseases 4. Service capacity and access Red = index value 61-80. Orange = index value 41-60. Yellow = 21-40. White = no data available. *Tanzania: FM training is offered in the private sector affiliated to Aga Khan University. Government policy does not support FM in the public sector.

Figure 2, Map 1 displays countries in SSA with the differing presences of FM programmes per country. 10,19–54 Map 2 displays UHC per country in SSA.

### What are the major factors enabling FM in SSA? Support from key stakeholders

Support from government was identified as a crucial factor in the successful launch of FM within a country. 27,28,44,55,56 Clear government policies advocating for the adoption of FM within healthcare systems, including strategies for the training and deployment of FPs, were a significant enabler for the roll out of FM.^48^Conversely, opposition or ambivalence by governments posed a significant barrier to the successful adoption of FM.^21^Several authors described a process of engagement with government departments designed to build support for FM and define its scope in the local context. 57

Other influential stakeholders included university authorities, such as heads of faculties or departments, professional bodies, medical regulators, doctors from other specialties and academic FM departments. Collaboration between stakeholders was important for developing a common understanding of FM, by defining the roles of FPs and training outcomes and creating an environment in which FM can develop.^29^,^47^

### Recognition of FPs as specialists

The importance of official recognition of FPs as specialists was identified. Recognition as specialists was found to aid recruitment and ensure that FPs are renumerated to the same degree as other specialists who undertake postgraduate training.^19^,^27^,^36^,^40^,^47^,^48^,^55^,^58^,^59^ Obtaining recognition from the private sector for the specialist status of FPs was identified as difficult in several countries.^36^,^49^

### International Collaboration

Eighteen papers emphasised the role of international collaboration in supporting FP training programmes, particularly during their infancy.^15^,^21^,^22^,^27^,^28^,^33^,^40^,^48^,^55^,^60^–^69^ Primary Care and Family Medicine Network for sub-Saharan Africa (PRIMAFAMED) was important for enhancing regional cooperation and providing support to newly established training programmes.^14^,^27^,^56^,^60^ The possibility of a regional college of FPs is currently being investigated.^27^

Support from North American and European institutions included the provision of short courses and training, expertise in faculty development, the provision of staff, grants for programme development, programme review and feedback, sharing of educational resources, and peer-to-peer research support.^22^,^28^,^33^,^40^,^61^,^62^,^64^,^66^,^67^,^69^The value of long-term, trusted collaborations and honest communication was highlighted.^22^There was an acceptance that, although support from overseas faculty is welcome, it is important to plan for a transition to local faculty as more FPs are trained to take up these positions.^28^,^66^,^67^ It was recognised that partners often had differing motivation and goals and so developing an understanding of these factors was important.^55^

Several papers described the learning that FPs and FM trainees gained from attendance at international conferences or from experience of FM in settings where the specialty was better established.^15^,^33^,^40^,^60^,^65^ Such experiences could inspire FPs and trainees to adapt their practice and provide encouragement in the face of FM training and practice difficulties.^15^

### Dedicated FPs and trainees

Several sources highlighted the dedication of FPs and FM trainees to their profession and to their patients. Such individuals acted as advocates for FM and were viewed as role models to emulate.^19^,^64^,^66^,^70^,^71^

### What are the major factors limiting the successful roll out of FM in SSA?

#### Lack of defined FM role within healthcare system

A major limiting factor is the lack of a defined role of FM within the healthcare system.^10^,^21^,^24^,^35^,^41^,^46^,^56^,^57^,^65^,^72^–^78^The key reasons for this were identified as a lack of career pathway, inappropriate deployment, and lack of critical mass to be visible within the healthcare system.

Confusion over the role of FPs extended to policymakers, patients, other doctors, medical students and FPs.^24^,^32^,^35^,^41^,^46^,^74^,^75^

Many sources noted that there is an absence of a clear career path and a lack of consultant posts.^58^,^65^,^79^ Such problems contribute to reduced interest in applying for FP training and a ‘brain drain’ whereby FPs, frustrated by the lack of opportunities to advance their careers, take jobs in other countries or in other specialties or sectors.^21^ One paper found that most FPs work in other specialties for part of their careers.^25^ There was evidence that some FPs were working in managerial roles, either because of a lack of clinical positions or because their skills meant that they were viewed as a good fit for such positions.^57^,^75^ Identified as ‘wonder doctors' who have generalist skills that can fill gaps in specialist services and referral hospitals FPs are placed within vertical health care systems and tertiary hospitals rather than the district setting for which they have been trained.^14^,^41^,^56^,^72^ The inappropriate deployment of FPs reduces the positive impact of FM within PHC and the visibility of FM within the healthcare system.^10^

A common theme identified was that healthcare professionals view FM not as its own speciality but made up of components of other specialities, resulting in ambiguity of the role.^72^ Additionally, this view promotes conflict with other specialties as it encourages other doctors to see FM as a threat to their practice.^20^ Several sources mentioned other specialties being suspicious of, or looking down on, FM, fuelled by a lack of clearly defined roles and scope of practice for FPs.^21^,^35^,^46^

#### Lack of FM faculty

A shortage of FPs who can act as faculty within FM programmes was identified.^19^,^20^,^36^,^58^,^62^,^63^,^66^,^77^,^79^,^80^ It was found that, whilst graduating FPs were encouraged to work within FM faculty, they lack the necessary training to work as educators.^19^,^81^ An assumption identified amongst employers was that FP could act as an educator without formal training.^82^ Barriers to training in medical education included insufficient funding for course fees and perceived lack of institutional support. 56, 81

The shortage of FM faculty impacts FM training. Several papers identified shortages of staff with sufficient knowledge of FM to deliver training and provide supervision.^22^,^66^ As a result, FM trainees identified that they had inappropriate preparation for examinations.^19^ A further sequela identified was poor mentor-mentee relationships.^83^

Several FM courses require the completion of a research project. However, several papers identified that trainees lacked institutional FM supervision and that resources were not adequate for completion of research projects.^63^,^66^,^79^ As a result, research projects represented a limiting step in completion of degrees.^63^

#### Inappropriate training settings

Many FM training programmes were often based at major tertiary hospitals.^56^ Many authors recognised that such settings did not provide suitable preparation for FM practice as presentations to tertiary centres were unlikely to represent the patient mix seen in primary care.^19^,^57^,^69^,^80^,^84^ Some universities deliberately decided to establish training programmes in rural areas to better approximate the eventual place of practice.^57^ Others were investigating moving their FP training to peripheral locations.^48^ However, several issues arose from running programmes away from university campuses, such as providing suitable accommodation for trainees and the risk that service providers would be prioritised over trainees' educational needs. 20,80 In addition, some authors considered that many trainees preferred to train in urban settings.^56^,^57^

#### High rates of trainee attrition

Several papers identified a significant discrepancy between the number of trainees entering programmes and those graduating.^64^,^75^ This was due to two main reasons: bottlenecks in training and high dropout rates.^21^,^36^,^67^,^75^ A contributing factor to bottlenecks was the difficulty in completing research projects.^67^,^79^ Reasons for the discontinuation of training included limited financial resources to pay trainees salaries, low pass rates in final examinations, and poor quality and levels of commitment amongst applicants.^21^,^36^,^64^,^81^,^83^ FM trainees were often expected to take on a significant clinical workload, seeing many patients daily. It was sometimes difficult for them to prioritise their educational needs or achieve a healthy work-life balance.^19^,^39^,^72^,^83^ The wages that FM trainees could earn from the clinical work associated with their training were sometimes insufficient for daily living expenses.^85^

#### Lack of FM in undergraduate curriculum

Undergraduate medical students were found to have limited exposure to FM as a specialty.^21^,^23^,^32^,^56^,^57^,^73^–^75^,^84^,^86^ If they existed at all, undergraduate placements in FM tended to be short and at the end of clinical training.^57^,^73^,^84^ The lack of exposure furthered misconceptions about the speciality. Two studies identified that medical students believe that FPs are the same as General Practitioners (GPs).^75^,^86^ Another consequence of limited exposure to FM was that few students intended to specialise in the discipline.^23^,^31^,^32^,^73^,^75^ An exception is Stellenbosch University which offers undergraduate medical students a year-long clerkship at a rural hospital, with supervision and teaching delivered by FPs.^38^

#### Lack of critical mass

The number of FPs deployed in SSA remains small.^37^,^87^ Reasons for this included high dropout rates from training programmes, limits on the number of training places resulting from a paucity of available trainers and financial constraints, lack of funded FP consultant and registrar roles, and loss of FPs to the private sector. 21,36,39,56,58,59,65,75,79 As a result, there are competing demands on FPs' time, and they often face difficulties in managing various responsibilities, which can result in burnout.^76^ The small number of FPs means that their impact is limited and there are few role models to encourage new trainees into the specialty.^56^ FPs often work in isolation, resulting in a lack of colleagues to discuss professional matters with.^46^ A further consequence, is that a limited number of FPs within the HCS limits the impact that the specialty intends to achieve.^88^,^89^

## Discussion

FM can strengthen PHC and improve UHC coverage. This has been recognised by an increasing number of countries in SSA who have acknowledged the potential contribution of FPs in strengthening PHC systems by formally recognising the discipline on a par with other specialties, creating training programmes and including FM within health system policies and strategies.

The major factors limiting FM programmes in SSA is the lack of a defined role, scope of practice and deployment of FPs within the healthcare system. National support from key healthcare stakeholders and international collaboration that is country specific and sustainable are important in strengthening FM programmes.

Despite the World Organisation of Family Doctors (WONCA) definition of FM and Rustenburg consensus , there is a lack of clarity about the discipline.^12^,^90^ Whilst the Astana Declaration of 2018 declared the need for strengthening PHC to achieve UHC, it failed to acknowledge which healthcare professionals could support this.^91^ A unified, international, or regional specific, definition of FM could aid the development of policy and healthcare systems that hold a clear role for FPs and integrate FM into the healthcare systems. This clarity could provide the structure to recruit and retain FPs, create consultant posts, and produce sufficient faculty for the development of undergraduate and postgraduate medical education. As a result of these structural changes , a critical mass of FPs could be achieved that can significantly impact PHC.

National support from key healthcare stakeholders, such as national ministries of health, increases the ease with which FM programmes are established. A clear, unified definition of the role of FM within the healthcare system will aid stakeholders to have a clear understanding of where FPs can be effectively deployed to support PHC. Further collaboration between stakeholders would better enable the development of FM programmes. The long term support of key individuals was identified as beneficial. Thus, changes in governments or in government personnel could have a significant negative impact if new postholders did not continue to prioritise the development of FM. To maintain support from stakeholders ad-vocates for FM need to engage with health system stakeholders on an ongoing and institutional level basis, so changes in individual personnel will be less likely to impact changes in policy.

Regional and international collaborators play an important role in supporting the development of FM. Multiple international institutions have supported the development of FM programmes in collaboration with government departments. However, as identified, this support must be adapted to the needs of the local setting as Global North FM programmes differ to those in SSA. An example of effective, sustainable, and country specific international collaboration is LeBoHa (The Lesotho, Boston Health Alliance). LeBoHa has developed a FM Speciality training programme, built on a longstanding relationship to develop a decentralised, non-university-based model with FM trainees based at rural-district hospitals to aid the retention of local doctors.^28^ A south-south network facilitated by PRIMAFAMED supports twinning of FM programmes between established South African programmes and developing programmes, giving regional specific support. On a wider level, PRIMAFAMED acts as a vital forum for sharing best practice and supporting FM development throughout SSA. 27 Given the considerable resource limitations faced by many countries in the region, and the commonalities that exist in FM practice across SSA, sharing of educational resources and pooling of expertise should be encouraged as far as possible.

The number of FPs in SSA remains small. As a result, the profession has failed to reach a critical mass and have a significant impact within PHC. A lack of critical mass reduces the visibility of FM within the healthcare system, limiting exposure to medical students, junior doctors and to maintaining political support. Additionally, without enough FPs to act as FM faculty there is a risk that training programmes are unsustainable. Stakeholders should capitalise on existing momentum in developing FM programmes and seek international support in conjunction with a defined country specific role of FM.

There is a high rate of attrition during FM training. Several difficulties facing trainees were identified; heavy clinical workload, poor remuneration, a lack of sufficient trainers to provide high-quality supervision, inadequate preparation for exams, inadequate preparation and support for research projects, and competition for educational opportunities from other specialties. Further research is needed to identify the key causative factors for the loss of FPs during training. Comparisons could be undertaken between specialties within similar healthcare systems that are able to recruit, train and retain postgraduate specialist doctors.

Evidence from medical undergraduates suggests that FM is not a popular specialty, attributed to a low exposure to FM and misconceptions concerning the specialty. Given the accepted need for many more generalist doctors in SSA to strengthen PHC in the region, there is a need to reorientate medical curricula to embed FM much more deeply into undergraduate training. Early exposure to FM is likely to improve the acceptability of the profession to trainees and increase trainee numbers. WONCA have committed to supporting the WHO and the United Nations to achieve the aims of the Astana Declaration by securing access to PHC with FPs in every community in the world, with each medical school having a department of primary care.^91^ Stellenbosch University already integrates FM into undergraduate medical education, with students able to complete the final year of studies in a district hospital under the supervision of a FP.^38^,^84^ Further development of such schemes could encourage undergraduates to pursue family medicine.

### Limitations

Papers were included only if they were published in a peer-reviewed journal. There may be important perspectives contained in other published literature, such as government reports. In addition, papers were included only if written in English. This is likely to bias the results towards countries where English is in common use and against Francophone and Lusophone SSA countries. Many of the papers included were written by individuals with postgraduate FM training or working in academic FM. Such individuals are likely to have a commitment to the specialty, which could influence their objectivity. The search was limited to 2015 onwards. Whilst this enabled current strengths and limitations to the development of FM programs to be identified it could have excluded previous papers that discuss the development of FM in SSA.

## Conclusion

FM can be key in strengthening PHC and enabling UHC in SSA. FM in SSA has expanded in recent years, and this paper has identified the major factors that have enabled FM to become established. However, the lack of a defined role for FM and its position within the healthcare system prevents the effective deployment of FPs. The standardisation of the role of FM and the implementation of undergraduate and postgraduate FM programmes with national and international collaboration could enable FM to reach a critical mass and realise its full potential in strengthening PHC in SSA. Steps are required to increase the proportion of trainees that successfully complete training and to ensure that those that do are deployed into appropriate posts in district health systems. In countries where FM has not yet been adopted, long-term engagement with relevant stakeholders will be required to build support for introducing the discipline.

## References

[1] World Health Organisation, Organisation for Economic Co-Operation and Development, and The World Bank (2018). Delivering Quality Health Services: A Global Imperative.

[2] The World Bank (2016). Universal health coverage in Africa: a framework for action.

[3] World Health Organisation and the International Bank for Reconstruction and Development / The World Bank (2017). Tracking universal health coverage: 2017 global monitoring report.

[4] Sadr-Azodi N (2019). Following the 2001 Abuja Declaration of committing 15 percent government expenditure on health, is Africa making progress towards universal health coverage? Master in Health Economics and Pharmacoeconomics (UPF Barcelona School of Management).

[5] Global Work Force Alliance and the World Health Organisation (2013). A Universal Truth: No Health without a workforce.

[6] World Health Organisation (2016). Global strategy on human resources for health: workforce 2030.

[7] World Health Organisation (2019). Primary health care on the road to universal health coverage: 2019 monitoring report.

[8] Starfield B, Powe N R, Weiner J R, Stuart M, Steinwachs D, Scholle S H (1994). Costs vs quality in different types of primary care settings. JAMA.

[9] World Health Organisation (2008). The world health report 2008: primary health care: now more than ever.

[10] Moosa S, Peersman W, Derese A, Kidd M, Pettigrew LM, Howe A (2018). Emerging role of family medicine in South Africa. BMJ Global Health.

[11] Flinkenflögel M, Sethlare V, Cubaka V M, Makasa M, Guyse A, De Maeseneer J (2020). A scoping review on family medicine in sub-Saharan Africa: practice, positioning and impact in African health care systems. Human Resources for Health.

[12] Mash R, Reid S (2010). Statement of consensus on Family Medicine in Africa. African Journal of Primary Health Care and Family Medicine.

[13] Flinkenflögel M, Essuman A, Chege P, Ayankogbe O, De Maeseneer J (2014). Family medicine training in sub-Saharan Africa: South-South cooperation in the Primafamed project as strategy for development. Family practice.

[14] Mash R, Howe A, Olayemi O, Makwero M, Ray S, Zerihun M (2018). Reflections on family medicine and primary healthcare in sub-Saharan Africa. BMJ Global Health.

[15] Larson PR, Chege P, Dahlman B, Gibson C (2017). Future of family medicine faculty development in sub-Saharan Africa. Family Medicine.

[16] Naidoo K, Schaefer R, Jenkins L S, Von Pressentin K B (2020). The evolving role of family physicians during the coronavirus disease 2019 crisis: An appreciative reflection. African Journal of Primary Health Care and Family Medicine.

[17] Motlhatlhedi K, Maotwe K, Bogatsu Y, Tsima B (2020). Coronavirus disease 2019 in Botswana: Contributions from family physicians. African Journal of Primary Health Care and Family Medicine.

[18] Fatusin B B, Odewale M A, Oseni T I, Agbede R O (2020). The role of the family physician in the fight against Coronavirus disease 2019 in Nigeria. African Journal of Primary Health Care and Family Medicine.

[19] Mbuka D O, Tshitenge S, Setlhare V, Tsima B, Adewale G (2016). New family medicine residency training programme: Residents' perspectives from the University of Botswana. African Journal of Primary Health Care and Family Medicine.

[20] Ogundipe R M, Mash R (2015). Development of Family Medicine training in Botswana: Views of key stakeholders in Ngamiland. African Journal of Primary Health Care and Family Medicine.

[21] Gossa W, Wondimagegn D, Demeke Mekonnen W E, Abebe Z, Fetters M D (2016). Key informants' perspectives on development of Family Medicine training programs in Ethiopia. Advances in Medical Education and Practice.

[22] Franey C, Evensen A, Bethune C, Zemenfes D (2016). Emergence of family medicine in Ethiopia: an international collaborative education model. Education for Primary Care.

[23] Lawson H J, Essuman A (2016). Country profile on family medicine and primary health care in Ghana. African Journal of Primary Health Care and Family Medicine.

[24] Mohamoud G, Mash B, Merali M, Orwa J, Mahoney M (2018). Perceptions regarding the scope of practice of family doctors amongst patients in primary care settings in Nairobi. African Journal of Primary Health Care and Family Medicine.

[25] Momanyi K, Dinant G-J, Bouwmans M, Jaarsma S, Chege P M (2020). Current status of family medicine in Kenya; family physicians' perception of their role. African Journal of Primary Health Care and Family Medicine.

[26] Malope S, Nkholongo E, Shaw K, Penti B, Schumacher R, Markuns J (2016). Development of a family medicine specialty training programme in Lesotho. The Lancet Global Health.

[27] Von Pressentin K B, Besigye I, Mash R, Malan Z (2020). The state of family medicine training programmes within the Primary Care and Family Medicine Education network. African Journal of Primary Health Care & Family Medicine.

[28] Bryden B, Bryden M, Steer-Massaro J, Malope S (2021). Family Medicine Training in Lesotho: A Strategy of Decentralised Training for Rural Physician Workforce Development. Frontiers in Medicine.

[29] Christians F (2020). Country profile-Primary healthcare and family medicine in Namibia. African Journal of Primary Health Care and Family Medicine.

[30] Yakubu K, Hoedebecke K, Pinho-Costa L, Popoola O, Okoye I (2017). A qualitative study of young Nigerian family physicians' views of their specialty. South African Family Practice.

[31] Ojo O, Egunjobi A, Fatusin A, Fatusin B, Adeyemo A (2018). A systematic review of the literature on the specialty preferences of Nigerian medical graduates: disparity between the literature and reality. South African Family Practice.

[32] Tanimu T S, Michael G C, Ibrahim A, Grema B A, Mohammed A A (2017). Awareness of family medicine discipline among clinical medical students of Bayero University, Kano, Nigeria. South African Family Practice.

[33] Flinkenflögel M, Ogunbanjo G, Cubaka V K, De Maeseneer J (2015). Rwandan family medicine residents expanding their training into South Africa: the use of South-South medical electives in enhancing learning experiences. BMC Medical Education.

[34] Robinson C (2019). Primary health care and family medicine in Sierra Leone. African Journal of Primary Health Care & Family Medicine.

[35] Saidiya C N (2020). The attitude and perceptions of doctors at Letaba Hospital towards family medicine: A qualitative study. South African Family Practice.

[36] Mash R, Von Pressentin K (2017). Family medicine in South Africa: exploring future scenarios. South African Family Practice.

[37] Von Pressentin K B (2021). The new human resources for health policy supports the need for South African family medicine training programmes to triple their output. South African Family Practice.

[38] De Villiers M, Hoffie Conradie P, Van Schalkwyk S (2018). Teaching medical students in a new rural longitudinal clerkship: opportunities and constraints. Annals of Global Health.

[39] Mash R, Ogunbanjo G, Naidoo S, Hellenberg D (2015). The contribution of family physicians to district health services: a national position paper for South Africa. South African Family Practice.

[40] Rogers D (2017). Family medicine in a globally-connected world: a South African perspective and the RCGP International and Overseas Network. BJGP Ppen.

[41] Von Pressentin KB, Mash RJ, Baldwin-Ragaven L, Botha RPG, Govender I, Steinberg WJ (2018). The bird's-eye perspective: how do district health managers experience the impact of family physicians within the South African district health system? A qualitative study. South African Family Practice.

[42] Gaede B (2020). History of academic family medicine in South Africa-When did it start?. South African Family Practice.

[43] Von Pressentin KB, Mash RJ, Baldwin-Ragaven L, Botha RPG, Govender I, Steinberg WJ (2018). The perceived impact of family physicians on the district health system in South Africa: a cross-sectional survey. BMC Family Practice.

[44] Mohamed K, Hunskaar S, Abdelrahman S, Malik E (2015). Telemedicine and e-learning in a primary care setting in Sudan: the experience of the Gezira Family Medicine Project. International Journal of Family Medicine.

[45] Ratanzi R, Gaede B M (2020). Family medicine in Tanzania: Seize the moment. African Journal of Primary Health Care & Family Medicine.

[46] Besigye IK, Onyango J, Ndoboli F, Hunt V, Haq C, Namatovu J (2019). Roles and challenges of family physicians in Uganda: A qualitative study. African Journal of Primary Health Care & Family Medicine.

[47] Makasa M, Nzala S, Sanders J (2015). Developing family medicine in Zambia. African Journal of Primary Health Care & Family Medicine.

[48] Ngoma M, Nzala S (2016). Developing a family medicine postgraduate training program in Zambia. Family Medicine.

[49] Sururu C, Mash R (2017). The views of key stakeholders in Zimbabwe on the introduction of postgraduate family medicine training: A qualitative study. African Journal of Primary Health Care and Family Medicine.

[50] Akoojee Y, Robert Mash R M (2017). Reaching national consensus on the core clinical skill outcomes for family medicine postgraduate training programmes in South Africa. African Journal of Primary Health Care and Family Medicine.

[51] Du Plessis D, Kapp P A, Jenkins L S, Giddy L (2016). Postgraduate training for family medicine in a rural district hospital in South Africa: Appropriateness and sufficiency of theatre procedures as a sentinel indicator. African Journal of Primary Health Care & Family Medicine.

[52] Mash R, Malan Z, Von Pressentin K (2016). Strengthening primary health care through primary care doctors: the design of a new national Postgraduate Diploma in Family Medicine: report. South African Family Practice.

[53] Mohamed K, Hunskaar S, Abdelrahman S, Malik E (2017). Confidence in procedural skills before and after a two-year master's programme in family medicine in Gezira State, Sudan. Advances in Medicine.

[54] Maïga MM, Blouin Genest G, Couturier F, Stecko S, Rietmann M, Coulibaly MB (2021). The contribution of family medicine to community-orientated health services in Mali: A short report. African Journal of Primary Health Care & Family Medicine.

[55] Evensen A, Wondimagegn D, Ashebir DZ, Rouleau K, Haq C, Ghavam-Rassoul A (2017). Family medicine in Ethiopia: lessons from a global collaboration. The Journal of the American Board of Family Medicine.

[56] Kapoor V, Penner J, Rouleau K, Chege PM, Godoy-Ruiz P, Rodas J (2017). Evolution of Family Medicine in Kenya (1990s to date): a case study. South African Family Practice.

[57] Makwero M, Lutala P, McDonald A (2017). Family medicine training and practice in Malawi: History, progress, and the anticipated role of the family physician in the Malawian health system. Malawi Medical Journal.

[58] Hellenberg D, Williams F R, Kubendra M, Kaimal R S (2018). Strengths and limitations of a family physician. Journal of Family Medicine and Primary Care.

[59] Tiwari R, Mash R, Karangwa I, Chikte U (2021). A human resources for health analysis of registered family medicine specialists in South Africa: 2002–19. Family Practice.

[60] Setlhare V (2016). Family medicine in Denmark: are there lessons for Botswana and Africa?: opinion paper.. African Journal of Primary Health Care and Family Medicine.

[61] Blitz J, Edwards J, Mash B, Mowle S (2016). Training the trainers: beyond providing a well-received course. Education for Primary Care.

[62] Lakhani N A, Nwosu O (2017). Developing family medicine in Ethiopia. Education for Primary Care.

[63] Goodyear-Smith F (2018). Collaborative postgraduate training in family medicine and primary care: Reflections on my visit to South Africa. African Journal of Primary Health Care & Family Medicine.

[64] Mash R, Blitz J, Edwards J, Mowle S (2018). Training of workplace-based clinical trainers in family medicine, South Africa: before-and-after evaluation. African Journal of Primary Health Care and Family Medicine.

[65] Fasola O (2018). Igniting a Paradigm Shift in Family Medicine in Nigeria: Lessons From a Global Health Experience. Family Medicine.

[66] Toma G, Essuman A, Fetters M D (2020). Family medicine residency training in Ghana after 20 years: resident attitudes about their education. Family Medicine and Community Health.

[67] McGuire C M, Riffenburg K, Malope S, Jack B, Borba C P (2020). Mixed-methods evaluation of family medicine research training and peer mentorship in Lesotho. African Journal of Primary Health Care and Family Medicine.

[68] Mash R, Blitz J, Malan Z, Von Pressentin K (2016). Leadership and governance: learning outcomes and competencies required of the family physician in the district health system. South African Family Practice.

[69] Sanoe I, Beyan-Davies K, Anyango S, Ekwen G, Pierre J, Farley J (2021). The Role of Family Medicine Training in Addressing Workforce Challenges in Rural Liberia-Early Implementation Experience. Annals of Global Health.

[70] Mash R (2019). World Family Doctors Day 2019: Reflections from an African perspective. African Journal of Primary Health Care & Family Medicine.

[71] Mohamed K G, Hunskaar S, Abdelrahman S H, Malik E M (2019). Impact on core values of family medicine from a 2-year Master's programme in Gezira, Sudan: observational study. BMC Family Practice.

[72] Mash R (2016). The contribution of family medicine to African health systems. African Journal of Primary Health Care & Family Medicine.

[73] Essuman A, Lawson H, Nortey D, Gyakobo M, Ofori-Amankwah G, Ndanu TA (2017). Five years of family medicine undergraduate education in Ghana: a wake-up call!. Ghana Medical Journal.

[74] Fasola O, Alao A, Ibisola B, Odekunle I, Obimakinde A (2019). Knowledge and perception of Family Medicine among medical students at University of Ibadan, Nigeria. South African Family Practice.

[75] Omed Ali R, Nkabinde T, Ross A (2019). Knowledge of final-year medical students at the University of KwaZulu-Natal about family medicine, and long-term career choices. South African Family Practice.

[76] Wagner L, Pather M K (2019). Exploring resilience in family physicians working in primary health care in the Cape Metropole. African Journal of Primary Health Care & Family Medicine.

[77] Wondimagegn D, Cornelson B, Rouleau K, Janakiram P, Ghavam-Rassoul A, Rodas J (2016). Toronto Addis Ababa Academic Collaboration in Family Medicine: an overview of the dawn of family medicine in Ethiopia through an inter-institutional model. Annals of Global Health.

[78] Chu K (2020). The role of family physicians in emergency and essential surgical care in the district health system in South Africa. South African Family Practice.

[79] Ras T, Schweitzer B, Bresick G, Hellenberg D (2004). Training family physicians: A qualitative exploration of experiences of registrars in a family medicine training programme in Cape Town, South Africa. South African Family Practice.

[80] Jenkins L S, Von Pressentin K (2018). Family medicine training in Africa: Views of clinical trainers and trainees. African Journal of Primary Health Care and Family Medicine.

[81] Larson P R, Chege P, Dahlman B, Gibson C, Eveneson A, del Colon-Gonzalez M (2017). Current status of family medicine faculty development in sub-Saharan Africa. Family Medicine.

[82] Johnson B, Cayley WE, Nguyen BM, Larson P, del C Colon-Gonzalez M, Gibson C (2017). Faculty development in family medicine education: what is needed?. The Pan African Medical Journal.

[83] Yakubu K, Flinkenflögel M, Okoye I, Lodenyo MM, Popoola O, Mohamoud G (2016). Perceived competency deficits and challenges of family medicine trainees in sub-Saharan Africa. Education for Primary Care.

[84] Essuman A, Mash R, Besigye I, Flinkenflögel M (2017). Conference report: Undergraduate family medicine and primary care training in Sub-Saharan Africa: Reflections of the PRIMAFAMED network. African Journal of Primary Health Care and Family Medicine.

[85] Gossa W, Jones C, Raiculescu S, Melaku M, Kebebew E, Zerihun M (2019). Family Medicine Residents' Attitudes About Training in Ethiopia and the United States. Family Medicine.

[86] Hagemeister DT, Pal A, Naidoo N, Kristen U, Mokgosana N, Joubert G (2017). Undergraduate medical students' interest in specialising in Family Medicine at the University of the Free State, 2014. South African Family Practice.

[87] Chinhoyi R L, Zunza M, Von Pressentin K B (2018). The impact of family physician supply on district health system performance, clinical processes and clinical outcomes in the Western Cape Province, South Africa (2011–2014). African Journal of Primary Health Care and Family Medicine.

[88] Mash R, Dyers R E (2015). How far does family physician supply correlate with district health system performance?. African Journal of Primary Health Care and Family Medicine.

[89] Mash R J, Von Pressentin K B, Esterhuizen T M (2017). Examining the influence of family physician supply on district health system performance in South Africa: An ecological analysis of key health indicators. African Journal of Primary Health Care and Family Medicine.

[90] Bentzen BG, Bridges-Webb C, Carmichael L, Ceitlin J, Feinbloom R, Metcalf D (1991). The role of the general practitioner/family physician in health care systems: a statement from WONCA [statement].

[91] American Academy of Family Physicians (2020). The declaration of Astana and what it means for the global role of NAPCRG and WONCA. Annals of Family Medicine.

